# Psychological symptoms, quality of life and dyadic relations in family members of intensive care survivors: a multicentre, prospective longitudinal cohort study

**DOI:** 10.1186/s13613-025-01420-8

**Published:** 2025-01-20

**Authors:** Sumeet Rai, Dale M. Needham, Rhonda Brown, Teresa Neeman, Krishnaswamy Sundararajan, Arvind Rajamani, Rakshit Panwar, Mary Nourse, Frank M. P. van Haren, Imogen Mitchell, for the PRICE study investigators

**Affiliations:** 1 School of Medicine and Psychology, Australian National University, Canberra, Australia; 2 Intensive Care Unit, Canberra Hospital, Canberra Health Services, Canberra, Australia; 3 John Hopkins University School of Medicine and School of Nursing, Baltimore, MD USA; 4 Research School of Psychology, Australian National University, Canberra, Australia; 5 School of Psychology, University of New England, Armidale, NSW Australia; 6 Biological Data Science Institute, College of Science, Australian National University, Canberra, Australia; 7 Intensive Care Unit, Royal Adelaide Hospital, Adelaide, Australia; 8 Faculty of Health and Medical Sciences, The University of Adelaide, Adelaide, Australia; 9 Intensive Care Unit, Nepean Hospital, Kingswood, Sydney, Australia; 10 Nepean Clinical School, University of Sydney, Kingswood, Sydney, Australia; 11 Intensive Care Unit, John Hunter Hospital, New Lambton, Australia; 12 School of Medicine and Public Health, University of Newcastle, Newcastle, Australia; 13 Intensive Care Unit, St. George Hospital, Kogarah, Sydney, Australia

**Keywords:** Post-intensive care syndrome-family, Intensive care, Psychology, Quality of life, Dyads

## Abstract

**Background:**

There is scarce literature evaluating long term psychological or Quality of Life (QoL) outcomes in family members of ICU survivors, who have not experienced invasive ventilation. The objective was to compare long-term psychological symptoms and QoL outcomes in family members of intubated versus non-intubated ICU survivors and to evaluate dyadic relationships between paired family members and survivors.

**Methods:**

Prospective, multicentre cohort study among four medical-surgical ICUs in Australia. Adult family members of ICU survivors and family-survivor dyads had follow-up assessments (3 and 12 months after ICU discharge), using Impact of Event Scale-Revised; Depression, Anxiety Stress Scales-21; EQ-5D-5L. Dyadic relationships examined associations of psychological symptoms or QoL impairments.

**Results:**

Of 144 family members (75% female, 54% partners/spouses) recruited, 59% cared for previously intubated survivors. Overall, 83% (110/132) of eligible family members completed ≥ 1 follow-up. In family members of intubated vs non-intubated survivors, clinically significant psychological symptoms (PTSD/depression/anxiety) were reported by 48% vs 33% at 3-months (p = 0.15); and 39% vs 25% at 12-months (p = 0.23). Family self-rated their QoL with a mean score of 83 (SD 13) on a visual analogue scale (range 0–100), and > 30% reported problems in pain/discomfort or anxiety/depression domains at 12-months. Family members were more likely to have persistent psychological symptoms of PTSD [OR 4.9, 95% CI (1.47–16.1), p = 0.01] or depression [OR 14.6, 95% CI (2.9–72.6), p = 0.001]; or QoL domain problems with pain/discomfort [OR 6.5, 95% CI (1.14–36.8), p = 0.03] or anxiety/depression [OR 3.5, 95% CI (1.02–12.1), p = 0.04], when the paired survivor also reported the same symptoms.

**Conclusions:**

Almost one-third of the family members of ICU survivors reported persistent psychological symptoms and QoL problems at 12-months. There was a noticeable dyad effect with family members more likely to have persistent symptoms of PTSD, depression, and problems in QoL domains when the paired ICU survivors experienced similar symptoms. The family members of non-intubated ICU survivors had an equal propensity to develop long-term psychological distress and should be included in long-term outcome studies. Future recovery intervention trials should be aimed at ICU family-survivor dyads.

*Trial registration* ACTRN12615000880549

**Supplementary Information:**

The online version contains supplementary material available at 10.1186/s13613-025-01420-8.

## Introduction

Family members often play a key part in the recovery of ICU survivors. However, a patient’s intensive care unit (ICU) stay can be emotionally traumatic for family members and may be associated with long-lasting symptoms of acute stress, anxiety, or anticipatory grief [1–3]. The term post-intensive care syndrome-family (PICS-F) was created to describe potentially long-lasting symptoms of post-traumatic stress disorder (PTSD), depression, and anxiety in family members of ICU survivors [1, 4].

The existing literature on long-term psychological impacts on family/caregivers of ICU survivors substantially varies in terms of the population studied (bereaved/non-bereaved family); timing of assessments (90 days, 6 months, 12 months, or longer); and assessment outcomes (caregiver burden, strain, psychological well-being, emotional distress or specific psychological symptoms) [5–7]. These studies demonstrating psychological symptoms or strain after discharge have largely evaluated outcomes of family members who have cared for invasively ventilated ICU survivors.

Additionally, due to their shared experiences, ICU survivors and family members may influence each other emotionally in dyad (paired) relationships [8, 9]. Previous studies of psychological outcomes in pairs of family members and ICU survivors have compared varied and different outcomes between family members (caregiving burden, strain, emotional distress, and psychological symptoms) and ICU survivors (psychological outcomes) [10–12]. These studies have also focused on family members of survivors who required invasive ventilation or evaluated outcomes in specific sub-groups of ICU survivors (sepsis, ARDS, chronic critically ill) [13, 14].

There is scarce literature evaluating long term psychological or Quality of Life (QoL) or dyad outcomes in family members of ICU survivors, who have not experienced invasive ventilation.

The Psychological stRess in Intensive CarE survivors (PRICE) study was designed to understand and compare the psychological and QoL outcomes of adult ICU survivors managed with and without invasive ventilation [15]. In this a priori designed sub-study, we compared psychological symptoms in non-bereaved family members of intubated vs. non-intubated ICU survivors at 3- and 12-months and sought to understand dyadic relationships between pairs of family members and ICU survivors.

## Materials and methods

PRICE was a multicentre prospective cohort study conducted in four tertiary hospitals in Australia from July 2015 until July 2019. The study was approved by the relevant local human research ethics committees and was registered (ACTRN12615000880549). The trial protocol, eligibility criteria, statistical analysis plan, and psychological and QoL outcomes of the ICU survivors were previously published [15, 16].

In accordance with the sub-study protocol, we enrolled family members of a mixed group of intubated (invasive ventilation > 24 h), and non-intubated (non-invasive ventilation and/or inotropic/vasopressor support) ICU patients, who survived to ward discharge. To assess new onset psychological symptoms after an ICU admission, survivors with pre-existing psychiatric disorders were excluded (detailed inclusion and exclusion criteria provided in Supplementary File 1). Family members were excluded if the ICU survivor did not consent to participate or if the patient died prior to the subsequent follow-up. ICU survivors and family members provided written, informed consent to participate. Pairs of family members and ICU survivors (dyads) were assessed longitudinally at 3- and 12-months (after ICU discharge) via post (mail). A trained research staff member attempted to contact participants by phone if there was no response to two study letters. Study results were reported using the Strengthening the Reporting of Observational Studies in Epidemiology guidelines [17]

### Study objectives

The primary objective was to compare psychological symptoms and QoL in family members of intubated vs. non-intubated ICU survivors at 3 and 12 months. The secondary objective was to explore the potential impact of dyadic relationship between ICU survivors and family members on the family members’ psychological symptoms and QoL problems.

### Measurement instruments

Validated, self-reported questionnaires were administered. Participants completed the Impact of Event Scale-Revised (IES-R) [18]; Depression, Anxiety Stress Scales-21 (DASS21) [19] at baseline, and 3 and 12 months after discharge from ICU. QoL was assessed using the EQ-5D-5L at follow-up [20] (details of assessment tools in Supplementary File 2). The IES-R (PTSD symptoms) was initially used in only the family members, while the EQ-5D-5L was initially used only in ICU survivors, but a protocol amendment permitted the measures to be administered for dyad comparison and analysis, to a majority of the subset of ICU survivors and family members, respectively.

### Statistical analysis

A detailed statistical analysis plan was published [15]. Based on the sub-study protocol, family members of 152 consenting patients/ICU survivors (recruited to the main study), were followed from baseline (ICU discharge) to 3 and 12 months. Data were included in the planned analyses if one or more follow-up assessments were completed.

Demographics and baseline characteristics were summarised using means and standard deviations (S.D.), medians and 25%–75% quartiles (interquartile ranges) for continuous measures, and frequencies (percentages) for categorical measures.

The primary outcome was presence of clinically significant psychological symptoms at the time of follow-up. Using published cut-off values for the scales, clinically significant symptoms were defined as a mean score ≥ 1.6 on IES-R (PTSD symptoms) [21, 22] and/or at least moderate severity for depression (≥ 14) or anxiety (≥ 10) using the DASS [23]. EQ-5D-5L dimensions were dichotomized as normal vs any problems in individual QoL dimensions. A priori comparisons between family members of intubated versus non-intubated survivors were used to evaluate the association of invasive ventilation on the family member’s outcomes. Generalised linear mixed effects models were used to compare the presence of clinically significant symptoms between the groups and follow-up time (3 and 12 months). A log linear mixed model, with group (intubated vs. non-intubated) and follow-up time (3 and 12 months) as fixed effects and family members as random effects, was used to compare screening tool scores between the groups at both follow-up times.

The secondary analysis explored the correlation between psychological symptoms and QoL problems in ICU survivors and their family members via their dyadic relationship, using a generalised mixed effect model analysis, with family members nested within the family unit, and with the family unit as a random effect. Dyad (family member and ICU survivor) pairs were included in the analysis, only if a complete set of follow-up observations, using the same assessment tool in both cohorts, was available at the specified time point. To determine the impact of individual symptoms on family members, dyadic modelling included the presence of similar psychological symptoms or QoL problems in ICU survivors at the time of follow-up.

Pearson’s chi-square test and Fisher’s exact test were used to compare categorical data, and the Wilcoxon rank sum test was used for continuous variables. Measures of association in the regression analysis were reported as odds ratio (OR) with 95% confidence intervals (CI). No imputation was conducted for incomplete or missing data (from loss to follow-up or non-response to an item). All data analysis was conducted using R statistical software version 4.4.0 and R studio, version 2023.12.1.402 for Windows [24, 25]. p < 0.05 was considered to be statistically significant.

## Results

**Fig. 1:**
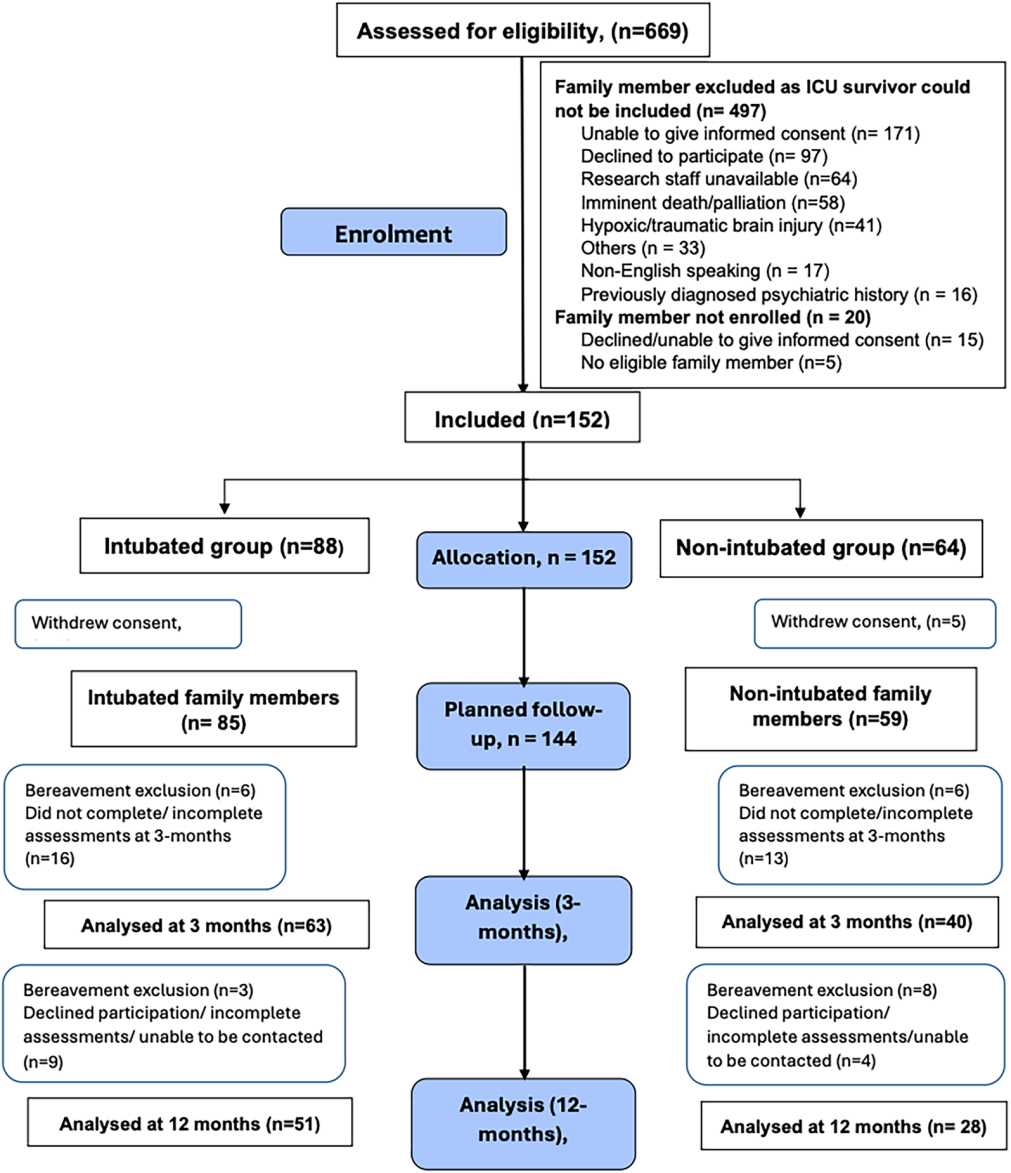
Consort diagram for family members. *ICU* intensive care unit

Paired family members were recruited for 152 survivors, of which 8 (5%) family members withdrew consent before any follow-up was commenced (Fig. 1). Of the 144 family members remaining, 108 (75%) were female, 78 (54%) were partners or spouses and 85 (59%) were caring for survivors who had been intubated (Table 1). Family members were caring for survivors, who had been predominantly admitted to the ICU for medical reasons (63%) and spent a median 4.3 [IQR 2.2–9.0] days in ICU and a median 19.1 [IQR 10.4–38.6] days in hospital. After excluding family members of subsequently deceased ICU patients from 3 [12 (8%)] and 12 months [11 (8%)] follow-up, there were 132 and 121 eligible family members at 3 and 12 months, respectively (Fig. 1). Of the total eligible family members, 83% (110/132) completed ≥ 1 follow-up, with data analysed for 103 family members and 97 dyads at 3-months, and 80 family members and 74 dyads at 12-months.

**Table 1:** Baseline family characteristics stratified by group of ICU patients

Characteristic	Intubated group (n = 85)	Non-intubated group (n = 59)
Female	68 (80%)	40 (68%)
Family relationship		
Partner/spouse	45 (53%)	33 (56%)
Child	28 (33%)	18 (31%)
Others^a^	12 (14%)	8 (14%)
Family co-habitating with patient	51/78 (65%)	41/58 (71%)
Patient characteristics		
Male	56/84 (67%)	28/57 (49%)
Medical admission	57/84 (68%)	32/57 (56%)
Chronic comorbidities^b^	28/84 (33%)	30/57 (53%)
APACHE III score	68 (32)	57 (17)
Delirium	28/83 (34%)	2/57 (4%)
ICU LOS (days)	7 [4.7–11.8]	2 [1.7–2.7]
Hospital LOS (days)	20 [12.0 −50.0]	17 [8.5–28.6]

Data presented as n (%) or mean (standard deviation) or median [interquartile range]

*APACHE* Acute Physiology and Chronic Health Evaluation, *LOS* length of stay

^a^Others (parent, grandparent, grandchild, sibling, friend)

^b^Chronic comorbidities: based on APACHE (organ system insufficiency or immunocompromised state)

### Psychological symptoms at follow-up

Among family members, clinically significant psychological scores for one or more symptoms (PTSD, depression, anxiety) were present in 42% (43/103) at 3-months and 34% (27/80) at 12-months follow-up. In family members of intubated vs non-intubated groups, clinically significant psychological scores were observed in 48% vs 33% at 3-months (p = 0.15); and 39% vs 25% at 12-months (p = 0.23) respectively (Table 2). Presence of clinically significant psychological symptoms and psychological assessment scores in the two groups of family members are detailed below.

**Table 2:** Clinically significant psychological symptoms between groups

Psychological symptoms	3 months				12 months			
	Intubated N = 63	Non-intubated N = 40	O.R (95% C.I.)	P value	Intubated N = 51	Non-intubated N = 29	O.R (95% C.I.)	P value
Any symptoms^^^	30 (48%)	13 (33%)	3.0 (0.68–13.4)	0.15	20 (39%)	7 (25%)	2.8 (0.52–15.4)	0.23
PTSD	29 (46%)	11 (28%)	2.2 (0.92–5.11)	0.08	20 (39%)	7 (26%)	1.8 (0.66–5.15)	0.24
Depression	9 (14%)	5 (13%)	1.2 (0.36–3.77)	0.80	8 (16%)	2 (7%)	2.4 (0.50–12.3)	0.29
Anxiety	6 (10%)	4 (10%)	0.95 (0.25–3.59)	0.94	4 (8%)	3 (11%)	0.7 (0.15–3.42)	0.67

^^^Any (≥ 1) clinically significant psychological symptoms for PTSD/depression/anxiety in family members

Data presented as n (%). O.R. (95% C.I.): Odds Ratio with 95% confidence intervals

For presence of each clinically significant symptom, a generalised linear mixed effects model was used with group and follow-up time (3 or 12 months) as fixed effects (with interaction). P-values and 95% C.I. are unadjusted for multiple comparisons

#### PTSD

**Fig. 2:**
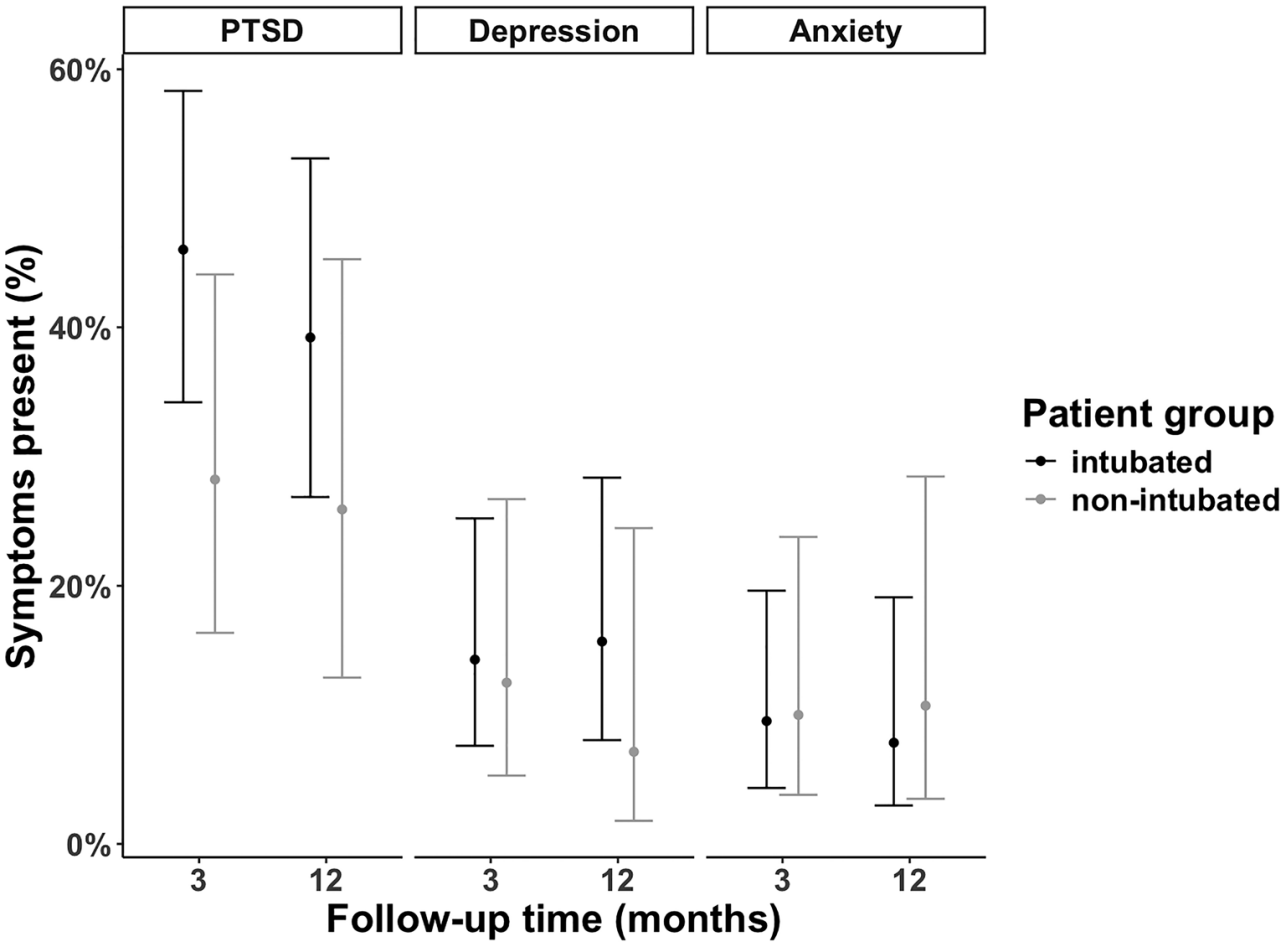
Clinically significant psychological symptoms in family members. Percentages and error bars for individual symptom from a generalised linear mixed effects model for clinically significant symptoms with follow-up time and group as fixed effects (with interaction). *PTSD* post-traumatic stress disorder

Clinically significant symptoms were seen in 39% and 35% of the family members at 3- and 12-month follow-up, respectively. Clinically significant PTSD symptoms were reported by a greater proportion of family members of the intubated vs. non-intubated groups at both follow-up points, however these differences were not statistically significant (Table 2, Fig. 2). Family members of the intubated group had significantly higher mean IES-R scores at 3-months (p = 0.03) but not at 12-months (p = 0.06) when compared to the non-intubated group (*Supplementary Table 1*).

#### Depression and anxiety

Clinically significant symptoms in the family members at 3- and 12-month follow-up were as follows: depression (14% and 13%) and anxiety (10% and 9%) respectively. There was no difference in the presence of clinically significant symptoms for depression or anxiety (Table 2, Fig. 2) or DASS scores for depression and anxiety (Supplementary Table 1) between the family members of the intubated vs. non-intubated groups at either follow-up point.

### Quality of life (QoL) at follow-up

**Fig. 3:**
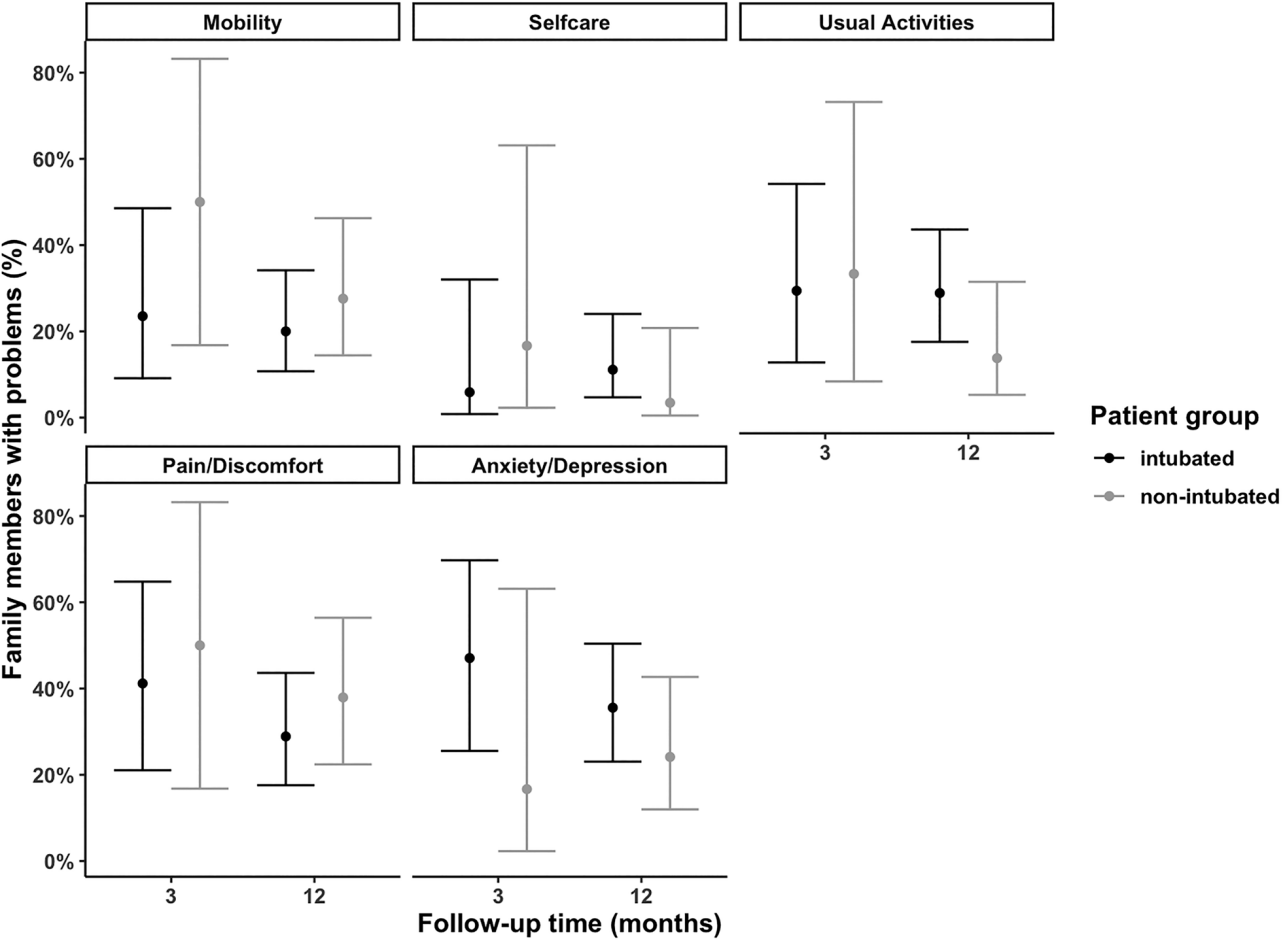
Problems in EQ5D domains reported by family members. Percentages and error bars for individual symptom from a generalised linear mixed effects model for clinically significant symptoms with follow-up time and group as fixed effects (with interaction)

Due to a late protocol amendment, only 23 eligible family members completed the EQ-5D-5L at 3 months and these data were included only in the dyad analysis. At 12 months, 74 family members completed EQ-5D-5L assessments and almost one-third reported problems in the pain/discomfort (32%) or the anxiety/depression (31%) domains, nearly one-quarter (23%) had problems with mobility and usual activities and 8% had problems with self-care (Fig. 3). At 12-months, mean (SD) values for QoL using the EQ-5D Visual Analogue Scale (VAS) was 83 (13) and for the EQ-5D-5L utility index was 0.94 (0.09). There was no difference in impairments/problems in the QoL domains (Supplementary Table 2), VAS or utility scores (Supplementary Table 1) between the family members of intubated vs. non-intubated groups.

### Dyadic relationship

Dyadic analysis at 3 months did not reveal strong association between the presence of symptoms of depression or anxiety in the family members when the same symptoms were analysed in the paired ICU survivors (Supplementary Table 3). Dyad analysis for depression at 12 months showed that family members were more likely to have symptoms when the paired survivor also had symptoms [OR 14.6, 95% CI (2.9–72.6), p = 0.001]. No dyad association of anxiety was observed at 12-months (Supplementary Table 3). For PTSD, analysis of the 61 dyads with completed IES-R at 12 months, showed a significant association of clinically significant symptoms in family members when paired ICU survivors also had symptoms [OR 4.9, 95% CI (1.47–16.1), p = 0.01]. Dyad analysis of QoL showed a strong association between family members reporting problems in the pain/discomfort [OR 6.5, 95% CI (1.14–36.8), p = 0.03] or anxiety/depression [OR 3.5, 95% CI (1.02–12.1), p = 0.04] domains when the paired survivor also reported matched problems, but this association was not detected in other QoL domains (Supplementary Table 4).

### Loss to follow-up

Overall, 23/144 (16%) family members were lost to follow-up at 3 and 12 months. A majority of family members lost to all follow-up cared for an intubated survivor and they were adult children of the survivors, but otherwise they did not differ in baseline characteristics or assessment scores, compared to those who completed follow-up (Supplementary Table 5). Of the 29 family members who did not complete the 3-month assessments, six later completed the 12-month follow-up and four were excluded from 12-month follow-up due to bereavement.

## Discussion

In this prospective longitudinal study from 4 Australian ICUs, more than one-third of the family members of ICU survivors reported persistent psychological symptoms and almost one-third reported QoL problems with pain/discomfort and anxiety/depression at 12-months. Similarly, more than one-third of family members reported persistent symptoms of PTSD at 3- and 12-month follow-up. Although a greater proportion of family members of intubated survivors reported psychological symptoms, there was no difference in clinically significant psychological symptoms or QoL problems, when compared to the family members of the non-intubated survivors. Family members were more likely to report persistent psychological symptoms of PTSD, depression, and QoL problems with pain/discomfort and anxiety/depression at 12 months when the paired ICU survivors experienced similar symptoms.

Family members are typically the informal caregivers of ICU survivors after discharge, which can lead to disruption of their lives and can contribute to psychological distress [2, 7, 26]. Our findings of psychological symptoms in non-bereaved family members of adult ICU survivors are broadly consistent with prior literature [12, 27–29]. Previous reviews have excluded family members of the non-intubated ICU survivors [30]. In our study, we included family members of the non-intubated ICU survivors and found a non-significant trend for psychological symptoms at 12-months being greater in family members of ICU survivors who were intubated compared to those who were non-intubated. It is possible that the non-significant results were due to the small group sizes, or they may have been influenced by other factors: for example, the greater incidence of delirium in intubated patients or the greater incidence of chronic co-morbidities in the non-intubated group (53% vs 32%). Despite this, the results suggest that the family members of non-intubated survivors also experienced stressors and/or had a propensity to develop long-term psychological distress.

Specifically, more than one-third of our study cohort reported persistent symptoms of PTSD at 3- and 12-month follow-up, consistent with the prior literature [10, 31, 32]. However, the presence of PTSD symptoms in family members in our study was almost five times higher than the Australian population normed values of 5.6% [33], and also higher than our previously published findings of PTSD symptoms in the ICU survivor (11–13%) cohort [16]. We did not investigate the reason for this difference, but it is likely that caregivers experienced acute stress as a result of the trauma of a loved one’s life being endangered, their involvement in complex decision making and taking on the role of surrogate decision-maker [11, 26]. Our dyad analysis may contribute to this understanding, as it indicates that family members of ICU survivors with PTSD symptoms were more likely to experience PTSD symptoms themselves at 12-months. The results also show that long-term depression and anxiety were prevalent in family of ICU survivors with no differences in symptoms when related to the intubation status of the patient. Our results indicate that the family members experienced more substantial distress at 12 months than Australian population normed values for depression (4.9%) and anxiety (3.8%) [33]. However, persistent depression lasting up to 12-months after discharge from the ICU in our study is lower than values reported in literature (21–43%) [12, 30, 34]. There is scant comparative literature on persistent anxiety in family members of ICU survivors at 12 months [30]. Persistent anxiety lasting up to 12-months after ICU discharge in our study is lower than that reported previously in literature (15% to 73%), largely limited to 3-months follow-up [35–37]. These differences in depression and anxiety rates may be due to higher (moderate or worse) levels of symptoms assessed in our study or difference in screening tools or participant samples from different continents or follow-up periods.

Over one-fifth of family members also reported problems with specific QoL domains at 12-months, and almost one-third reported problems with pain/discomfort or anxiety/depression. Our results are consistent with the previous literature reporting persistent (90 days to 1-year) decrements in mental health domains of QoL in family members of ICU survivors [38, 39].

Our study findings shed further light on the emotional interconnectedness of ICU survivors and their caregivers, within dyadic relationships such that the presence of psychological symptoms (PTSD or depression) or QoL problems were strongly linked in the matched pairs. Prior research using fewer dyads have shown similarities in psychological distress symptoms experienced by ICU survivors and caregivers over time [40] and higher caregiver burden at 3-months, when paired ICU survivors had adverse physical or psychological outcomes [41]. Although our study was not designed to test dyad dynamics using an actor-partner interdependence model (APIM) [13], it does add to the growing literature on understanding dyad experiences and adds to the debate on the role for targeting dyadic interventions in critical care recovery [42, 43].

We present our study’s limitations along with future research directions to guide advancements in PICS-F. First, as a sub-study, it was not powered to compare the family members of intubated vs non-intubated survivors, so these findings may be exploratory in nature. Additionally, a higher-than-expected mortality in the non-intubated group, and the resultant exclusion of bereaved family members, led to an imbalance in the groups at long-term follow-up. Hence, it is possible that differences between groups or dyads might have gone undetected. Future research should be adequately powered to compare outcomes and dyad interactions between family members of the intubated vs the non-intubated ICU survivors. Second, the study attrition over 12 months was 16%. However, over 80% of the family members completed at least one assessment time point and there was no difference in the baseline characteristics of study participants relative to those lost to follow-up. Our attrition rate was similar to rates reported in prior follow-up studies of family members of ICU survivors [12, 41] and may be attributed to survey fatigue, higher psychological burden, or potentially competing time commitments in caregiving roles. Subsequent studies employing a mixed methods design should aim to characterize participation patterns and identify risk factors, barriers, and facilitators that influence engagement in studies on PICS-F. Third, we did not collect specific characteristics of family members (e.g. age, psychological comorbidity, educational attainment), the extent of caregiving provided to the survivor, or social support received by the family member, which limits further investigation into these factors. Expanding future research to examine post-ICU experiences, including the rehabilitation experience, follow-up medical care, and social support, could yield valuable insights into PICS-F. This approach would shift focus beyond ICU factors alone, helping to identify additional influences during the recovery process that impact psychological and quality of life outcomes for patients and their families.

The strengths of this study include the prospective, multicentre design that recruited family members from a mixed group of ICU survivors, which permit the results to be generalized. Further, the paired dyadic analysis of family members and ICU survivors adds to the literature on emotional interdependence of the ICU survivor and their caregivers. Finally, the study inclusion of family members of non-intubated survivors provides insight into the long-term psychological effects of family members who provide caregiving to an increasing population of ICU survivors.

## Conclusions

Almost one-third of the family members of ICU survivors reported persistent psychological symptoms and QoL problems at 12-months. There was a noticeable dyad effect with family members more likely to have persistent symptoms of PTSD, depression, and problems in QoL domains when the paired ICU survivors experienced similar symptoms. The family members of non-intubated ICU survivors had an equal propensity to develop long-term psychological distress and should be included in long-term outcome studies. Future recovery intervention trials should be aimed at ICU family-survivor dyads.

## Supplementary Information

Supplementary Material 1.

## Data Availability

The datasets used and/or analysed during the current study are available from the corresponding author on reasonable request.

## Abbreviations

QoL: Quality of Life
ICU: Intensive care unit
PTSD: Post Traumatic Stress Disorder
PICS-F: Post-Intensive Care Syndrome-Family
IES-R: Impact of Event Scale-Revised
DASS21: Depression, Anxiety Stress Scales 21
APACHE: Acute Physiology and Chronic Health Evaluation
LOS: Length of stay
VAS: Visual Analogue Scale
APIM: Actor-Partner Interdependence Model
