# Supplementary material for: Psychological symptoms, quality of life and dyadic relations in family members of intensive care survivors: a multicentre, prospective longitudinal cohort study
Source: Ann Intensive Care. 2025 Jan 20;15:14. doi: 10.1186/s13613-025-01420-8 (PMC11746989; doi:10.1186/s13613-025-01420-8)
Supplement: Supplementary file 1 — Supplementary Material 1. [file 13613_2025_1420_MOESM1_ESM.docx]

**Supplementary materials**

- **Supplementary File 1:** Methods: Inclusion and Exclusion criteria
- **Supplementary File 2:** Details of assessment tools
- **Supplementary Table 1:** Comparative scores for screening tools
- **Supplementary Table 2:** Problems in QoL domains in family members at 12-months
- **Supplementary Table 3:** Dyad analysis for association of psychological symptoms
- **Supplementary Table 4:** Dyad analysis for association of problems in Quality of Life (QoL) domains
- **Supplementary Table 5:** Baseline characteristics of family members, stratified by loss to follow-up

**Supplementary file 1: Methods - Inclusion/Exclusion criteria**

**Adult family member of a consenting ICU patient participating in the main long-term follow-up PRICE study, with the following criteria.**

**Inclusion criteria:**

*Adult patients (18 years or older at the time of ICU admission)*

**A. Intubated ICU survivor:**

- Intubated and mechanically ventilated for more than 24 hours
- Stayed in the ICU for more than 72 hours

**B. Non-intubated ICU survivor:**

- Not intubated during current ICU stay
- Received inotropic/vasopressor support and/or non-invasive ventilation during ICU stay.

**Exclusion criteria (for ICU survivor)**

- Prior history of a psychiatric disorder (e.g. psychotic disorders, chronic PTSD)
- Imminent death/palliative care patient (unlikely to be alive at follow-up)
- Suspected acute primary brain lesion that may result in global impairment of consciousness or cognition, such as traumatic brain injury, intracranial haemorrhage, stroke or hypoxic brain injury

**Exclusion criteria (for survivor/family member)**

- Unable to give informed consent prior to hospital discharge.
- Non-English-speaking background

**Supplementary File 2: Details of assessment tools**

**IES-R**: 22 item screening tool (each item score ranges from 0-4) assessing symptoms of PTSD. The IES-R has good sensitivity and specificity, using a combined mean score threshold of >1.6 (Weiss 2004), when compared a clinician administered PTSD diagnostic tool (Bienvenu et al 2013).

**DASS21:** 21-item scale examining the presence and severity of symptoms of depression, anxiety, and stress, with seven items examining each symptom state. Participants provided self-ratings using a 4-point scale (0-3). Published cut-off scores for the subscales (higher scores indicating worse symptoms) indicate the extent to which the symptoms are clinically relevant relative to normed values for each state.

**EQ-5D-5L**: participants described their status across five dimensions: mobility, self-care, usual activities, pain, and anxiety/depression. Each dimension was scored from 1-5 (1 = no problems to 5 = extreme problem) and individual dimension scores were converted to a single utility score using Australian normative data. The utility score can range from a negative value (i.e. a state worse than death) to zero (representing death) to 1 (representing full health). Participants also rated their overall health using a visual analogue scale (VAS) ranging from 0 to 100, with a score of 100 indicating the best possible health.

**Supplementary table 1: Comparative scores of family members**

| **Screening tool** | **3-months** | | | **12-months** | | |
| --- | --- | --- | --- | --- | --- | --- |
|  | **Intubated**  **N=63** | **Non-intubated**  **N=40** | **P value** | **Intubated**  **N=51** | **Non-intubated**  **N=29** | **P value** |
| **IES-R** | 1.5 (0.2) | 0.9 (0.2) | **0.03*** | 1.2 (0.2) | 0.7 (0.2) | 0.06 |
| **DASS Depression** | 2.9 (0.5) | 2.2 (0.6) | 0.37 | 2.2 (0.5) | 1.5 (0.5) | 0.35 |
| **DASS**  **Anxiety** | 1.2 (0.3) | 1.2 (0.3) | 0.89 | 0.9 (0.2) | 1.1 (0.4) | 0.68 |
| **EQ-5D VAS** |  |  |  | 82 (3) | 83 (3) | 0.81 |
| **EQ-5D-5L**  **utility score** |  |  |  | 0.95 (0.01) | 0.93 (0.02) | 0.42 |

IES-R: Impact of Event Scale-Revised; DASS: Depression Anxiety Stress Scale

VAS: Visual Analogue Scale

Estimated means (standard errors) are presented for a) IES-R and DASS: derived from a log linear mixed model, with individual scores as the independent variable, follow-up time and group as fixed effect and family member as a random effect b) EQ5D: derived from linear mixed model, with 12-month score as independent variable and patient group as fixed effect.

P-values are unadjusted for multiple comparisons.

**Supplementary table 2: Problems in QoL domains in family members at 12-months**

|  | **Intubated**  **N=45** | **Non-intubated**  **N=29** | **p-value^^^** |
| --- | --- | --- | --- |
| **Mobility** | 9 (20%) | 8 (28%) | 0.4 |
| **Self-care** | 5 (11%) | 1 (3%) | 0.4 |
| **Usual Activities** | 13 (29%) | 4 (14%) | 0.13 |
| **Pain/**  **Discomfort** | 13 (29%) | 11 (38%) | 0.4 |
| **Anxiety/**  **Depression** | 16 (36%) | 7 (24%) | 0.3 |

Data presented as n (%) representing any problems in the EQ-5D-5L domains.

^Fisher’s exact test

**Supplementary Table 3: Dyad analysis for association of psychological symptoms**

|  | **3 months** | | |  | | **12 months** | | |
| --- | --- | --- | --- | --- | --- | --- | --- | --- |
| **Psychological symptoms** | **n** | **Odds Ratio**  **(95% C.I.)** | **p-value** | **n** | **Odds Ratio**  **(95% C.I.)** | | **p-value** |  |
| **PTSD** | 23 | 4.0 (0.61-26.1) | 0.15 | 61 | 4.9 (1.47-16.1) | | 0.01* |  |
| **Depression** | 97 | 1.8 (0.64-5.22) | 0.26 | 75 | 14.6 (2.9-72.59) | | 0.001* |  |
| **Anxiety** | 97 | 1.5 (0.47-4.66) | 0.51 | 75 | 1.3 (0.23-6.94) | | 0.79 |  |

Association between presence of symptoms in family members and matched symptoms in ICU survivor. Odds ratios were estimated using a generalised mixed effect model with each psychological symptom in family member as response (presence/absence), and matched symptom in survivor and time as fixed effects.

**Supplementary table 4: Dyad analysis for association of problems in Quality of Life (QoL) domains**

| **QoL domains** | **Odds Ratio (95% C.I.)** | **p-value^^^** |
| --- | --- | --- |
| **Mobility** | 1.2 (0.2-3.18) | 0.78 |
| **Self-care** | 1.6 (0-2133) | 0.89 |
| **Usual Activities** | 1.7 (0.57-4.77) | 0.36 |
| **Pain/Discomfort** | 6.47 (1.14-36.8) | 0.03* |
| **Anxiety/Depression** | 3.52 (1.02-12.1) | 0.04* |

*Dyad analysis of QoL domains included 22 complete dyads at 3-months and 67 dyads at 12-month follow-up.*

Association between problems in QoL in family members and matched problem in ICU survivor. Odds ratios were estimated using a generalised mixed effect model for each problem in family member (presence/absence), with matched problem in survivor and time as fixed effects.

| **Supplementary table 5: Baseline characteristics of family members, stratified by loss to follow-up** | | | |
| --- | --- | --- | --- |
| **Characteristic** | **Lost to follow-up** | | **p-value^** |
|  | **No**  N = 121 | **Yes**  N = 23 |  |
| **Intubated** | 72 (60%) | 13 (57%) | 0.79 |
| **Female** | 89 (74%) | 19 (83%) | 0.36 |
| **Relationship** |  |  | 0.08 |
| Partner | 70 (58%) | 8 (35%) |  |
| Child | 34 (28%) | 12 (52%) |  |
| Others | 17 (14%) | 3 (13%) |  |
| **Family co-habitating with patient** | 82 (71%) | 10 (45%) | 0.05 |
| **Baseline IES-R** | 2.8 (2.2) | 2.3 (1.7) | 0.46 |
| **Baseline DASS21 Depress** | 7 (8) | 9 (10) | 0.62 |
| **Baseline DASS21 Anxiety** | 6 (7) | 8 (9) | 0.49 |
| Data presented as n (%) and mean (standard deviation) | | | |
| ^Associations between family member characteristic and lost-to-follow-up were assessed with Fisher’s exact test (intubation status, gender, relationship, cohabitation) or with a Wilcoxon rank sum test (IES-R, DASS21 scores). | | | |
